# Mobile Health, Disease Knowledge, and Self-Care Behavior in Chronic Kidney Disease: A Prospective Cohort Study

**DOI:** 10.3390/jpm11090845

**Published:** 2021-08-27

**Authors:** Yi-Chun Tsai, Pei-Ni Hsiao, Mei-Chuan Kuo, Shu-Li Wang, Tzu-Hui Chen, Lan-Fang Kung, Shih-Ming Hsiao, Ming-Yen Lin, Shang-Jyh Hwang, Hung-Chun Chen, Yi-Wen Chiu

**Affiliations:** 1 Department of Nephrology, Kaohsiung Medical University Hospital, Kaohsiung Medical University, Kaohsiung 807, Taiwan; 920254@kmuh.org.tw (Y.-C.T.); mechku@cc.kmu.edu.tw (M.-C.K.); 1080421@kmuh.org.tw (M.-Y.L.); sjhwang@kmu.edu.tw (S.-J.H.); chenhc@kmu.edu.tw (H.-C.C.); 2 School of Medicine, College of Medicine, Kaohsiung Medical University, Kaohsiung 807, Taiwan; 3 Department of Internal Medicine, Division of General Medicine, Kaohsiung Medical University Hospital, Kaohsiung Medical University, Kaohsiung 807, Taiwan; 4 Liquid Biopsy and Cohort Research Center, Kaohsiung Medical University, Kaohsiung 807, Taiwan; 5 Department of Nursing, Kaohsiung Medical University Hospital, Kaohsiung Medical University, Kaohsiung 807, Taiwan; 880212@kmuh.org.tw (P.-N.H.); 860007@kmuh.org.tw (S.-L.W.); 990238@kmuh.org.tw (T.-H.C.); 900191@kmuh.org.tw (L.-F.K.); 830230@kmuh.org.tw (S.-M.H.)

**Keywords:** mHealth, disease knowledge, self-care behavior, chronic kidney disease

## Abstract

Mobile health (mHealth) management is an emerging strategy of care for patients with chronic diseases. However, the effect of mHealth management on clinical outcomes of patients with chronic kidney disease (CKD) has not been well-studied. The aim of this study was to investigate the additional influence of mHealth on disease knowledge and self-care behavior in CKD patients who had received traditional education. We designed and developed a new healthcare mobile application, called iCKD, which has several major features, including home-based physiological signal monitoring, disease health education, nutrition analysis, medication reminder, and alarms and a warning system. Trained nurses interviewed patients with CKD using structured questionnaires of disease knowledge and self-care behavior. After propensity score matching, we analyzed 107 patients who used iCKD and traditional education, and 107 who received traditional education. The patients who used iCKD had higher disease knowledge scores than those who received traditional education. In multivariate analysis, iCKD was significantly and positively associated with disease knowledge scores. Patients with high education levels could have greater disease knowledge through using mHealth. There was no significant difference in total scores of self-care behavior between the two groups. In conclusion, mHealth can significantly increase disease knowledge in patients with CKD.

## 1. Introduction

Chronic kidney disease (CKD) is a global public health issue and is associated with high rates of morbidity and mortality. CKD has been reported to affect 14% of the US population and 12% of the Taiwanese population, contributing to a high burden on health-care systems [1,2]. CKD management is challenging, and includes prevention, early diagnosis, and the development of a thorough disease-management program [3,4]. Mobile health (mHealth) is an emerging strategy of care for patients with chronic diseases, such as diabetes mellitus (DM), hypertension, heart disease, stroke and asthma, that is accessible through mobile devices such as smartphones, tablets, or web-based portals [5]. mHealth has been shown to improve communication between healthcare professionals and patients and to facilitate monitoring bio-information at home and health behavior management, and to further promote the early detection of a worsening health status [6].

Managing CKD care is complicated due to its indolent course, many associated comorbidities, several therapeutic goals that need to be achieved, and poor prognosis. CKD patients commonly have a low awareness and limited understanding of their illness [7]. Accurate disease knowledge has been shown to play an important role in self-care and further self-management of chronic diseases [8], and CKD care programs may therefore increase the level of disease knowledge and improve self-care behavior, which in turn may lead to better clinical outcomes [9,10,11]. However, Gray et al. reported that disease knowledge remained limited with only small changes compared to the baseline after 1 year of educational interventions using traditional methods [12]. In addition, Yen et al. reported that educational interventions could result in short-term but not long-term increases in disease knowledge [13]. Consequently, it is important for health-care providers to develop new tools to improve health behavior. To date, the effect of mHealth education on disease knowledge and self-care behavior in patients with CKD has not been well-studied. Therefore, the aim of this study was to investigate the additional influence of mHealth combined with traditional health education on disease knowledge and self-care behavior compared to only traditional health education (face-to-face or paper-based) in patients with CKD.

## 2. Materials and Methods

### 2.1. Study Participants

**Figure 1:**
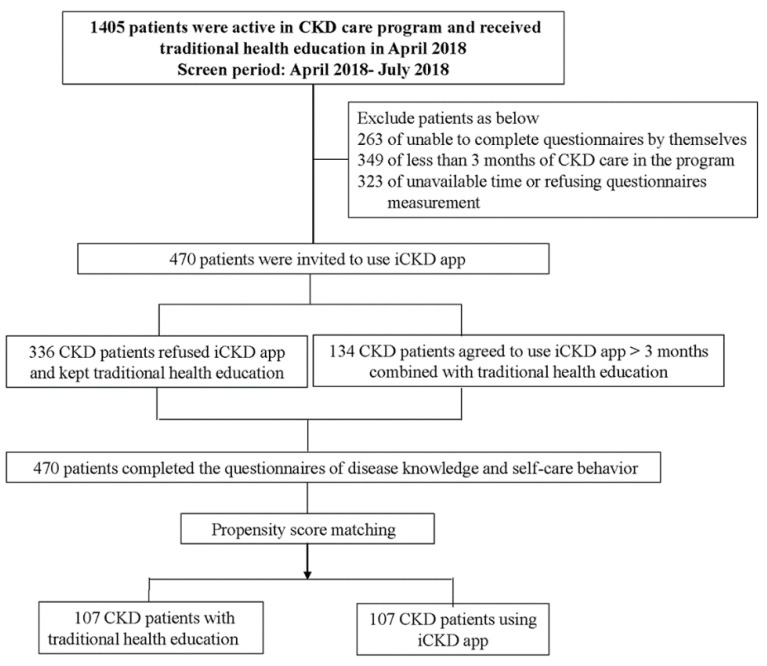
The flow chart of this study.

This study included patients with CKD stages 1–5 who were not receiving renal replacement therapy at Kaohsiung Medical University Hospital (KMUH) and participated in a CKD care program, which was conducted by a cross-disciplinary team, including clinical physicians, nursing staff, pharmacists, and nutritionists. The stages of CKD were defined according to the Kidney Disease Outcomes Quality Initiative (K/DOQI) guidelines. Estimated glomerular filtration rate (eGFR) was calculated using the four-variable Modification of Diet in Renal Disease Study equation as follows (stage 1 ≥ 90 mL/min/1.73 m^2^; stage 2 60–89 mL/min/1.73 m^2^; stage 3a 45–59 mL/min/1.73 m^2^; stage 3b 30–44 mL/min/1.73 m^2^; stage 4 15–29 mL/min/1.73 m^2^; stage 5 < 15 mL/min/1.73 m^2^) [14,15]. The contents of CKD traditional education included introducing knowledge of the kidney’s function and anatomy, symptoms, risk factors and complications of kidney disease, and interpretation of laboratory data of kidney disease. In addition, nutritional education was also given on eating healthily and what foods are limited due to renal disease. The frequency of traditional education was dependent on CKD stages (once every six months for CKD stages 1–2, once every three months for CKD stages 3–4, and once every month for CKD stage 5). A total of 1405 patients who were enrolled in the care program in April 2018 were scheduled for regular outpatient visits between April 2018 and July 2018 and invited to attend a study interview. The exclusion criteria in this study were being enrolled in the CKD care program for <3 months (*n* = 349), being unavailable or refusing to enter the study (*n* = 323) and being unable to complete the questionnaires by him/herself in daily life (*n* = 263). A total of 470 patients receiving traditional health education were invited to use the iCKD app. Finally, 134 agreed to use the iCKD app for 3 months at least combined with traditional health education (iCKD group), and the others refused to use the iCKD app because of the lack of a smartphone or inability to handle devices and kept traditional health education (non-iCKD group) (Figure 1). Then, these patients completed the questionnaires of disease knowledge and self-care behavior. The study protocol was approved by the Institutional Review Board of KMUH. Written informed consent was obtained from all patients, and all clinical investigations were conducted according to the principles expressed in the Declaration of Helsinki.

### 2.2. iCKD Development

**Figure 2:**
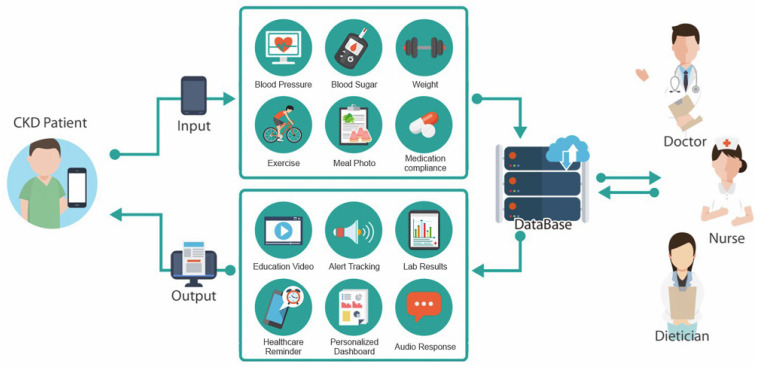
iCKD health management application platform.

Kaohsiung Medical University Hospital (KMUH) in southern Taiwan designed and developed the iCKD Healthcare Technology Platform, a digital learning and information service, in April 2013. The platform includes the iCKD health management application (iCKD app) and a collaborative management platform (Figure 2). The iCKD platform has ten major features, including alert tracking, outpatient visit status reminder, mobile health management, interpretation of examination results, tailored health education videos, nutritional feedback and analysis, care and treatment notifications, audio responses, a personalized dashboard, and integrated care quality analysis. Clinical physicians can use the collaborative management platform to remotely analyze and monitor a patient’s health status with self-recorded data via the personal dashboard. The healthcare quality indicators allow the related healthcare teams to assess the current disease status and provide timely feedback with online reminders.

### 2.3. Clinical Measurements

Information on socio-demographic and clinical characteristics such as age, sex, tobacco smoking (yes vs. no), alcohol consumption (yes vs. no), marital status, education level, current occupation, and co-morbidities were obtained from interviews with the patients and medical records at study interview. Marital status, including being married, single, divorced, or widowed, and working state, including currently working, no job, or retirement, were recorded. Hypertension was defined as having a history of the disease and taking antihypertensive medications before enrollment. Blood pressure was measured at both home and office. Heart disease was defined as having history of congestive heart failure, myocardial infarction, or ischemic heart disease. DM was defined as having a history of the disease, the use of anti-diabetic medications, or blood glucose values as defined in the American Diabetes Association criteria. The duration of CKD was calculated from self-reports and the initial diagnosis of CKD. The number of health education sessions was calculated between enrollment into the multidisciplinary CKD program and study interview. Blood and urine samples were taken after a 12-h fast for biochemistry studies on the same day as the study interview. Urine protein levels were measured using urine protein–creatinine ratio (PCR).

### 2.4. Disease Knowledge and Self-Care Behavior Measurement

We used the Perceived Kidney Knowledge Survey (PIKS) with a score range (SR) of 9–36 to measure disease knowledge in study patients [16]. PIKS contains nine questions, including the knowledge about the medications preserving renal function or causing renal injury, diet restriction in CKD, therapeutic goal of blood pressure control, renal replacement therapy, symptoms of CKD, renal function and its evaluation, and nephrology referral. The PIKS is a convenient tool to help care providers to gain a greater understanding of perceived kidney knowledge and improve clinical communication with patients with CKD [16].

We used the CKD Self-Care (CKDSC) scale to assess self-care behavior, which we previously validated for use in patients with CKD [17]. It is a 16-item questionnaire with five domains including diet control (SR: 4–20), medication adherence (SR: 5–25), blood pressure (SR: 2–10), exercise (SR: 3–15), and smoking habits (SR: 2–10).

### 2.5. Propensity Score Matching

Propensity score (PS) matching was performed to balance confounders between the comparisons of interest (iCKD usage vs. non-iCKD usage) and to minimize the confounding by indication resulting from nonrandom treatment study [18]. Using a logistic regression model, iCKD usage was accessed to estimate the propensity to receive the iCKD app for each participant based on potential confounders, including age, sex, socio-demographic characteristics (marital status, currently working, education level, and CKD duration), comorbidities (hypertension, heart disease, and DM), and renal function. We used PS-matched (1:1) analysis to match CKD patients who did or did not use iCKD app (Figure 1).

### 2.6. Statistical Analysis

Data were expressed as mean ± SD or median (25th, 75th percentiles) for continuous variables, and percentages for categorical variables. Non-normally distributed continuous variables were log-transformed to approximate normal distribution. The independent *t*-test or Mann–Whitney U analysis was used to compare two groups of continuous variables, and the chi-square test was used to evaluate differences in the distribution of categorical variables. Multivariate linear regression was used to evaluate correlations between the use of iCKD and disease knowledge and self-care behavior. Multivariate models were adjusted for all variables in Table 1 with *p*-value < 0.05 in the univariate analysis. To decrease selecting bias in the multivariable analysis, forward analysis with adjusting all variables in Table 1 was used. The interaction between iCKD usage and age, sex, renal function, and education level was tested in disease knowledge using univariate analysis of variance. Statistical analyses were conducted using SPSS version 22.0 for Windows (SPSS Inc., Chicago, Illinois). Graphs were drawn using Graph Pad Prism 5.0 (GraphPad Software Inc., San Diego CA, USA). Statistical significance was set at a two-sided *p*-value of ≤ 0.05.

## 3. Results

### 3.1. Characteristics of the Entire Cohort

We used PS matching to reduce selection bias of the study subjects, with 107 patients who used iCKD and 107 patients who did not use iCKD being entered into the final analysis (Figure 1). The mean duration of iCKD usage was 2.0 ± 0.9 years. Comparisons of clinical characteristics between the groups based on iCKD usage are shown in Table 1. The mean age of the 214 patients was 63.9 ± 11.5 years, 59.3% were male, and 83.6%, 12.6%, and 33.6% had hypertension, heart disease, and diabetes, respectively. Of all patients, 79.0% were married, 66.8% had graduated from senior high school or above, and 37.9% were currently working. The mean self-care and disease knowledge scores were 64.3 ± 10.1 and 23.6 ± 6.0, respectively. There were no significant differences in demographic and laboratory parameters between the patients who did and did not use iCKD. The patients who did not use iCKD received more traditional health education sessions than those who used iCKD. The patients who used iCKD had higher disease knowledge scores than those who did not use iCKD, but there was no significant difference in self-care scores between the two groups.

### 3.2. mHealth, Disease Knowledge and Self-Behavior

We further analyzed nine items of disease knowledge and five items of self-care behavior between the two groups (Table 2). Among these nine items of disease knowledge, the scores of blood pressure goal, treatment options if kidney function gets worse, symptoms of CKD, and functions of the kidney were higher in the patients who used the iCKD app than those who did not use. There were no significant differences in knowledge of medications that help the kidney, medications that can hurt the kidney, foods to avoid if kidney function is low, how kidney function is checked, or why a patient was sent to a kidney doctor between the two groups.

Among these five items of self-care behavior, patients who used the iCKD app had higher scores of blood pressure monitoring than those who did not use it. No significant differences in diet, exercise, smoking habits, or medication adherence were found between two groups.

To examine the correlation between iCKD usage and disease knowledge, we used linear regression analysis. In univariate analysis, disease knowledge was positively correlated with marriage (β = 3.02, *p* = 0.003), education level with senior high school or above (β = 4.69, *p* < 0.001), currently working (β = 2.12, *p* = 0.01), duration of CKD (β = 0.11, *p* = 0.03), and iCKD usage (β = 1.95, *p* = 0.02), but negatively correlated with age (β = −0.15, *p* < 0.001), and glycated hemoglobin level (β = −1.05, *p* = 0.01) (Table 3). After adjusting for these variables in Table 1 with *p* < 0.05 in univariate linear analysis, high disease knowledge scores were associated with a young age, an education level of senior high school or above, longer duration of CKD, lower glycated hemoglobin level, and iCKD usage. Additionally, iCKD usage was still significantly correlated with high disease knowledge scores in forward linear regression analysis (Table S1).

**Figure 3:**
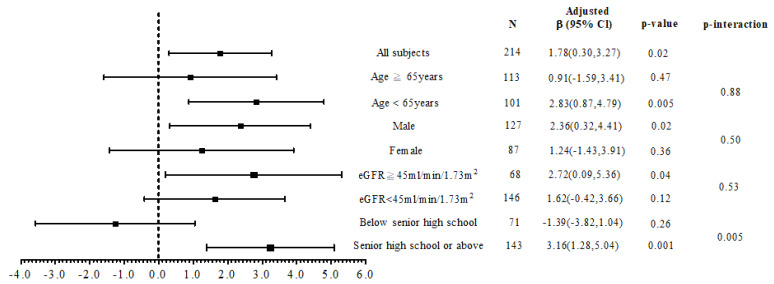
The association between mHealth usage and disease knowledge in subgroup analysis.

In order to analyze the interactions between iCKD usage and age, sex, renal function, and education level, the patients were stratified by an age of median (65 years), sex, eGFR of 45 mL/min/1.73 m^2^, and education level of senior in high school (Figure 3). The results showed that the patients who used iCKD had higher disease knowledge scores in those who were < 65 years of age (β = 2.83, *p* = 0.005), male (β = 2.36, *p* = 0.02), eGFR ≥ 45 mL/min/1.73 m^2^ (β = 2.72, *p* = 0.04), and education level of senior in high school or above (β = 3.16, *p* = 0.001). There was an interaction effect between iCKD usage and education level on disease knowledge (*p*-interaction = 0.005). However, there was no correlation between iCKD usage and self-care behavior scores in linear regression analysis.

### 3.3. Sensitivity Analysis

We analyzed all study subjects (*n* = 470) and stratified them according to iCKD usage (Table S2). In multivariate linear regression analysis with adjusting all variables in Table with *p* < 0.05 in univariate analysis, iCKD usage was significantly associated with high disease knowledge score (β = 1.18, *p* = 0.04, Table S3).

## 4. Discussion

We conducted this prospective study to evaluate the additional impact of mHealth on disease knowledge and self-care behavior in patients with CKD receiving traditional health education. Our results showed that the patients who used mHealth in their daily lives had greater disease knowledge than those who did not use mHealth. mHealth was positively and significantly correlated with disease knowledge, although there was no effect on self-care behavior. In addition, education level had an interaction effect on the relationship between mHealth and disease knowledge. Patients with high education level could have greater disease knowledge through using mHealth.

CKD care programs have been developed to help patients with CKD to learn about the potential risk factors for renal injury, and the diagnosis and treatment of CKD [19,20]. Compared with mHealth, paper-based or face-to-face traditional education is limited by the availability of providers, and it also therefore limits the knowledge and understanding of CKD care with regard to self-care behavior in daily life. In the current study, we found that the patients who used the iCKD app had higher scores of disease knowledge than those receiving traditional education. The mobile iCKD app provides knowledge and health information at any time and place through visual and audio formats of content delivery to allow the patient to engage with the learning material and reinforce knowledge, and it can help with understanding and inspire subsequent behavior changes [21,22].

We further analyzed nine items of disease knowledge in the patients who did and did not use the iCKD app and found that the patients who used mHealth had higher scores in knowledge of blood pressure goals, treatment options if kidney function gets worse, symptoms of CKD, and functions of the kidney, compared to those who did not use mHealth. The iCKD app provides tailored health education videos to help patients gain a greater understanding of the functions of the kidney, symptoms of CKD, and treatment options if kidney function gets worse. Alert tracking and audio responses can provide warnings and concise educational reminders to patients, reminding them to pay attention to biochemical risks, such as low hemoglobin level or hyperkalemia, and review previously learned materials. The iCKD app makes health education more convenient and efficient. Additionally, the app allows the patients to view their home-recorded blood pressure data with alert tracking and audio responses, thereby prompting them to develop regular blood pressure monitoring habits and be aware of blood pressure goals. However, there was no significant difference between the CKD patients who did and did not use the iCKD app in foods to avoid if kidney function is low, knowledge of medications that help the kidney, medications that can hurt the kidney, and how kidney function is checked. Our CKD patients usually knew which foods to avoid if kidney function is impaired after regular traditional education; however, the iCKD app did not have an additional effect on improving knowledge about renal nutrition (Table 2). Further, our platform lacks an immediate response to drug usage, and this may lead to insufficient knowledge of medications. Therefore, we may enhance functionality with regard to nutritional and medication feedback and analysis and interpreting examination results to improve their disease knowledge.

mHealth may face the potential challenges of a “digital divide” between people of different ages, education levels, and baseline self-efficacy [23]. Older CKD patients may have lower motivation and ability to use mobile health applications compared with younger CKD patients. In addition, CKD patients with high education levels might benefit more from mHealth for CKD care. Thus, we performed propensity score matching and subgroup analysis to diminish the interactional effect of age, sex, education level, and baseline renal function on the relationship between iCKD and disease knowledge. A positive correlation between disease knowledge and mHealth usage were found in patients with age < 65 years, who were male, had eGFR ≥ 45 mL/min/1.73 m^2^, and had a high education level, and there was an interaction effect between education level and mHealth usage on disease knowledge. We suggest that health-care providers could encourage CKD patients with high education levels to use mHealth to improve their disease knowledge to achieve better clinical outcomes. With regard to the barriers of using mHealth in CKD patients who are older or have low education levels, the iCKD app could be made easier to use by simplifying the interface system, enhancing the touch system or using a voice control system [24,25]. In addition, health-care providers could spend more time teaching these patients and repeat the evaluation of their ability to use mHealth. Furthermore, families of these patients also play an important role in CKD care. Health-care providers could invite young family members to join health-care programs and assist the patients to use mHealth.

Accurate disease knowledge has been associated with precise self-care behavior [8]. Among five items of self-care behavior, we found that patients who used the iCKD app had greater performance of blood pressure monitoring than those who did not use it. Patients can view their home-recorded blood pressure and submit them to health-care providers via the iCKD app. Previous studies have also reported a reduction in home-recorded blood pressure in CKD patients using mobile health applications compared to those using usual care [26,27]. However, we did not find a positive association between mHealth and total scores of self-care behaviors in patients with CKD. This may be due to the relatively short observation period. Knowledge needs to be internalized before it is converted into behavior, and patients may spend more time to learn and understand accurate knowledge before showing improved self-care behavior. In addition to having enough disease knowledge, motivation plays an important role in performing self-care well. Encouraging motivation to carry out good self-care behavior is not easy. Patients may be invited to join group meetings of health-care and medical decisions to establish the partnership between patients and health-care providers to promote their self-care behavior [28,29]. These steps may improve self-care and develop self-efficiency. Further longitudinal studies are needed to evaluate the long-term effect of mHealth on self-care behavior in patients with CKD.

Use frequency and duration can influence the impact of mHealth on the management of chronic disease [30]. However, the study conducted by Vehi et al. in type 1 and type 2 diabetic patients revealed an insignificant relationship between the use frequency of the Social Diabetes app (SDA) and glycemic control [31], meaning that higher-frequency use did not elucidate improved glycemic levels. This disassociation was probably the result of high uptake and engagement of mHealth in the beginning of contacting mHealth and the decline in use frequency later [32,33]. Continuous and consistent use of mHealth rather than higher frequency use might lead to improved clinical performance. In addition, providing mHealth to patient does not automatically equate to them engaging and using mHealth to manage their disease if patients have no active motivation, which was supported by the study conducted by Borelli et al. indicating that smokers unmotivated to quit were less likely to use mHealth materials [34]. Further study is necessary to examine the optimum frequency and consistency when using mHealth devices to manage CKD. How to enhance the motivation to engage and use mHealth remains an important subject of technology-based interventions of long-term CKD care.

There are several limitations in this study. Firstly, 72% of patients who used the iCKD app uploaded their home blood pressure or checked their laboratory data once a week at least for more than 3 months. Precise personal habits of mHealth use and engagement could not be evaluated because the iCKD app lacked a system to monitor the use frequency and duration, and this might affect the actual impact of the iCKD app on disease knowledge. The system will be upgraded in the future to evaluate the effect of the frequency of usage on disease knowledge or self-care behavior. Secondly, we measured disease knowledge and self-care behavior only after the intervention, and not at baseline. The aim of this study was to examine the additional impact of mHealth combined with traditional education on disease knowledge and self-care behavior compared to traditional education, not to investigate disease knowledge and self-care behavior before and after using the iCKD app. PS matching was used to lower the selection bias. Further prospective studies will be conducted to evaluate the dynamic impact of mHealth on disease knowledge and self-care behavior. Finally, questionnaires were used to examine disease knowledge and self-care behavior, and recall bias may have affected the results. Although patients who were unable to complete the questionnaires by themselves were excluded, the results might not be applicable to all populations.

## 5. Conclusions

CKD patients who received mHealth education had higher scores of disease knowledge than those who did not receive mHealth education. mHealth enhanced CKD knowledge but did not improve self-care behavior. Further longitudinal or randomized control trials are needed to evaluate the therapeutic impact of mHealth on clinical outcomes in patients with CKD.

## Figures and Tables

**Table 1:** The clinical characteristics of study subjects stratified by iCKD usage post propensity score matching.

Clinical characteristics	Entire Cohort N = 214	iCKD N = 107	Non-iCKD N = 107	*p*-Value
Demographics				
Age (years)	63.9 ± 11.5	63.5 ± 11.1	64.2 ± 11.9	0.67
Sex (male, %)	59.3	62.6	56.1	0.33
Smoke (yes, %)	25.7	26.2	25.2	0.87
Alcohol (yes, %)	10.7	12.1	9.3	0.50
Marital status (married, %)	79.0	83.2	74.8	0.13
Currently working (yes, %)	37.9	38.3	37.4	0.88
Education (senior high school or above, %)	66.8	70.1	63.6	0.31
Hypertension (yes, %)	83.6	84.1	83.2	0.85
Diabetes mellitus (yes, %)	33.6	35.5	31.8	0.56
Heart disease (yes, %)	12.6	13.1	12.1	0.83
Body mass index (kg/m^2^)	24.5 ± 4.4	24.3 ± 4.4	24.5 ± 3.8	0.78
Traditional health education (session)	19.7 ± 11.1	17.0 ± 10.7	22.5 ± 10.9	< 0.001
The duration of CKD (year)	11.3 ± 8.2	10.4. ± 8.9	12.1 ± 7.5	0.12
Systolic blood pressure (mmHg)	136 ± 17	137. ± 15	135 ± 18	0.25
Diastolic blood pressure (mmHg)	77 ± 10	77. ± 10	76 ± 10	0.46
Questionnaires				
Self-care score	64.3 ± 10.1	64.4 ± 9.4	64.1 ± 10.7	0.84
Disease knowledge score	23.6 ± 6.0	24.6 ± 6.6	22.6 ± 5.3	0.02
Laboratory parameters				
Blood urea nitrogen (mg/dL)	27.6 (19.5, 42.5)	26.4 (19.8, 43.4)	27.6 (18.1, 41.8)	0.39
eGFR (ml/min/1.73 m^2^)	36.0 ± 24.9	35.0 ± 23.5	37.0 ± 26.3	0.55
Hemoglobin (g/dL)	12.1 ± 2.2	12.2 ± 2.2	12.1 ± 2.1	0.67
Albumin (g/dL)	4.3 ± 0.4	4.3 ± 0.3	4.3 ± 0.4	0.34
Uric acid (mg/dL)	6.5 ± 1.6	6.5 ± 1.6	6.6 ± 1.5	0.88
Cholesterol (mg/dL)	174 ± 38	172 ± 38	177 ± 38	0.34
Triglyceride (mg/dL)	105 (78, 147)	107 (80, 146)	99 (76, 152)	0.29
Urine protein/creatinine ratio (mg/mg)	0.6 (0.2, 1.5)	0.7 (0.2, 1.6)	0.5 (0.2, 1.4)	0.35
Glycated hemoglobin (%)	5.8 (5.5, 6.3)	5.8 (5.5, 6.3)	5.9 (5.5, 6.3)	0.52

Data are expressed as number (percentage) for categorical variables and mean ± SD or median (25th, 75th percentile) for continuous variables, as appropriate. Abbreviations: eGFR, estimated glomerular filtration rate; CKD, chronic kidney disease.

**Table 2:** The items of disease knowledge and self-care behavior between CKD patients with and without iCKD usage post propensity score matching.

Questionnaires	Entire cohort N = 214	iCKD N = 107	Non-iCKD N = 107	*p*-Value
Disease knowledge				
Knowledge of medications that help the kidney	2.3 ± 1.0	2.4 ± 1.0	2.2 ± 0.9	0.26
Knowledge of medications that can hurt the kidney	2.5 ± 0.9	2.6 ± 0.9	2.4 ± 0.9	0.18
Knowledge of foods to avoid if kidney function is low	2.9 ± 0.8	2.9 ± 0.8	2.8 ± 0.8	0.60
Knowledge of blood pressure goal	2.9 ± 0.8	3.1 ± 0.8	2.7 ± 0.7	0.001
Knowledge of treatment options if kidney function gets worse	2.4 ± 1.0	2.5 ± 1.0	2.3 ± 0.9	0.04
Knowledge of symptoms of chronic kidney disease	2.3 ± 0.9	2.6 ± 0.9	2.1 ± 0.9	0.001
Knowledge of how kidney function is checked	2.7 ± 0.9	2.8 ± 1.0	2.6 ± 0.8	0.31
Knowledge of functions of the kidney	2.6 ± 0.9	2.7 ± 0.9	2.5 ± 0.8	0.01
Knowledge of why patient was sent to a kidney doctor	3.0 ± 0.8	3.1 ± 0.9	2.9 ± 0.7	0.25
Self-care behavior				
Diet	14.8 ± 3.6	14.7 ± 3.5	14.9 ± 3.8	0.76
Exercise	10.2 ± 4.1	10.2 ± 3.9	10.1 ± 4.3	0.95
Blood pressure monitoring	6.9 ± 2.6	7.4 ± 2.4	6.4 ± 2.7	0.005
Smoking habits	9.0 ± 2.2	8.8 ± 2.4	9.2 ± 2.0	0.21
Medication adherence	23.4 ± 2.7	23.2 ± 2.6	2.3.5 ± 2.9	0.44

Data are expressed as mean ± SD or median (25th, 75th percentile) for continuous variables Abbreviations: eGFR, estimated glomerular filtration rate; CKD, chronic kidney disease.

**Table 3:** The determinant of disease knowledge score in 214 CKD patients post propensity score matching.

	Disease Knowledge Score			
	Univariate		Multivariate	
	β(95% Cl)	*p*-Value	β(95% Cl)	*p*-Value
Clinical characteristics				
Age (per year)	−0.15(−0.22, −0.08)	<0.001	−0.09(−0.17, −0.01)	0.03
Sex (female vs. male)	−0.81(−2.47, 0.85)	0.34	−0.00(−1.68, 1.68)	0.99
Smoke (yes vs. no)	−1.72(−3.57, 0.14)	0.07	--	--
Alcohol (yes vs. no)	0.32(−2.32, 2.96)	0.81	--	--
Married status (yes vs. no)	3.02(1.06, 4.98)	0.003	1.71(−0.24, 3.67)	0.09
Current working (yes vs. no)	2.12(0.46, 3.78)	0.01	0.18(−1.65, 2.01)	0.85
Education (senior high school or above vs. below senior high school)	4.69(3.08, 6.30)	<0.001	3.22(1.46, 4.98)	<0.001
Hypertension (yes vs. no)	−0.43(−2.64, 1.78)	0.70	--	--
Diabetes mellitus (yes vs. no)	−1.61(−3.33, 0.10)	0.07	--	--
Heart disease (yes vs. no)	0.48(−1.98, 2.93)	0.70	--	--
Body mass index (per kg/m^2^)	−0.12(−0.32, 0.08)	0.23	--	--
Health education time (per session)	0.06(−0.02, 0.13)	0.13	--	--
CKD duration (per year)	0.11(0.01, 0.21)	0.03	0.11(0.02, 0.20)	0.02
iCKD usage (yes vs. no)	1.95(0.34, 3.56)	0.02	1.78(0.30, 3.27)	0.02
Blood urea nitrogen (per mg/dL)	0.03(−0.01, 0.07)	0.15	--	--
eGFR (per ml/min/1.73 m^2^)	−0.01(−0.05, 0.02)	0.44	--	--
Log-formed glycated hemoglobin	−1.05(−1.89, −0.22)	0.01	−0.90(−1.66, −0.13)	0.02
Hemoglobin (per g/dL)	−0.08(−0.46, 0.29)	0.66	--	--
Albumin (per g/dL)	1.25(−0.99, 3.49)	0.27	--	--
Uric acid (per mg/dL)	−0.25(−0.78, 0.28)	0.36	--	--
Cholesterol (per mg/dL)	0.01(−0.01, 0.03)	0.27	--	--
Log-formed triglyceride	−3.22(−6.78, 0.34)	0.08	--	--
Log-formed urine protein/creatinine ratio	−0.21(−1.64, 1.21)	0.77	--	--

Abbreviations: eGFR, estimated glomerular filtration rate; CKD, chronic kidney disease.

## Data Availability

The data presented in this study are available on request from the corresponding author. The data are not publicly available due to patients’ privacy.

## Supplementary Materials

The following are available online at https://www.mdpi.com/article/10.3390/jpm11090845/s1, Table S1. The determinant of disease knowledge score in 214 study subjects post propensity score matching using forward linear regression analysis. Table S2. The clinical characteristics of 470 study subjects stratified by CKD stages. Table S3. The determinant of disease knowledge score in 470 study subjects.
