# Supplementary material for: Mobile Health, Disease Knowledge, and Self-Care Behavior in Chronic Kidney Disease: A Prospective Cohort Study
Source: J Pers Med. 2021 Aug 27;11(9):845. doi: 10.3390/jpm11090845 (PMC8469557; doi:10.3390/jpm11090845)
Supplement: Supplementary file 1 [file jpm-11-00845-s001.zip › jpm-1328316-supplementary.pdf]

Table S1. The determinant of disease knowledge score in 214 study subjects post propensity score matching using forward linear regression analysis

	Forward<br>β(95%Cl)	P-value
Age (per year)	-0.15(-0.22,-0.08)	<0.001
Smoke (yes *v.s.* no)	-2.22(-3.90,-0.55)	0.01
Education (senior high school or above *v.s.* below senior high school)	3.68(2.06,5.31)	<0.001
Health education time (per session)	0.14(0.07,0.20)	<0.001
Heart disease (yes *v.s.* no)	2.43(0.19,4.68)	0.03
Log-formed glycated hemoglobin	-0.75(-1.49,-0.01)	0.05
iCKD usage (yes *v.s.* no)	2.36(0.88,3.83)	0.002

Abbreviations: CKD, chronic kidney disease

Forward linear regression analysis was adjusted for all variable in Table 1

Table S2. The clinical characteristics of 470 study subjects stratified by iCKD usage

	Entire Cohort N=470	iCKD N=134	Non-iCKD N=336	P-value
**Demographics**				
Age (year)	65.9±11.9	59.0±13.9	68.6±9.8	<0.001
Sex (male, %)	55.7	58.2	54.8	0.49
Smoke (yes, %)	21.7	23.1	21.1	0.63
Alcohol (yes, %)	10.4	11.2	10.1	0.73
Marriage (yes, %)	77.2	78.4	76.8	0.71
Occupation (yes, %)	28.7	47.8	21.1	<0.001
Education (senior high school or above, %)	53.2	75.4	44.3	<0.001
Hypertension (yes, %)	84.0	84.3	83.9	0.92
Diabetes mellitus (yes, %)	38.5	29.9	42.0	0.02
Heart disease (yes, %)	19.1	11.9	22.0	0.01
Body mass index (kg/m^2)	24.7±4.3	24.6±4.8	24.7±4.1	0.87
Health education times (session)	15.1±10.4	16.5±10.5	14.6±10.4	0.07
The time of CKD (year)	9.4±7.6	9.9±8.5	9.1±7.2	0.30
**Questionnaires**				
Self-care score	64.2±9.6	63.9±9.4	64.3±9.8	0.67
Disease knowledge score	22.7±5.7	24.9±6.2	21.8±5.2	<0.001
**Laboratory parameters**				
Blood urea nitrogen (mg/dl)	28.0(19.8,45.4)	23.6(17.7,41.1)	29.6(20.6,46.3)	0.05
eGFR (ml/min/1.73m^2)	34.1±23.0	38.7±26.4	32.2±21.3	0.01
Hemoglobin (g/dl)	11.9±2.0	12.3±2.2	11.8±1.9	0.004
Albumin (g/dl)	4.3±0.3	4.3±0.4	4.2±0.3	0.04
Uric acid (mg/dl)	6.6±1.5	6.5±1.5	6.6±1.6	0.53
Cholesterol (mg/dl)	174±37	174±37	174±38	0.86
Triglyceride (mg/dl)	108(79,163)	117(84,150)	108(77,165)	0.54
Urine protein/creatinine ratio (mg/mg)	0.6(0.2,1.5)	0.6(0.2,1.5)	0.6(0.2,1.5)	0.93
Glycated hemoglobin (%)	5.9(5.6,6.5)	5.7(5.4,6.2)	5.9(5.6,6.6)	0.001

Data are expressed as number (percentage) for categorical variables and mean±SD or median (25th, 75th percentile) for continuous variables, as appropriate.

Abbreviations: eGFR, estimated glomerular filtration rate

Table S3. The determinant of disease knowledge score in 470 study subjects

	Disease Knowledge score			
	Univariate		Multivariate (Forward)	
	β(95%Cl)	P-value	β(95%Cl)	P-value
**Clinical characteristics**				
Age (per year)	-0.14(-0.18,-0.09)	<0.001	-0.08(-0.13,-0.04)	<0.001
Sex (female *v.s.* male)	-0.64(-1.67,0.40)	0.23	--	--
Smoke (yes *v.s.* no)	-0.96(-2.22,0.28)	0.13	--	--
Alcohol (yes *v.s.* no)	0.36(-1.33,2.05)	0.67	--	--
Marriage (yes *v.s.* no)	1.03(-0.20,2.26)	0.09	--	--
Currently working (yes *v.s.* no)	2.24(1.11,3.36)	<0.001	--	--
Education (senior high school or above *v.s.* below senior high school)	4.00(3.03,4.97)	<0.001	2.63(1.63,3.63)	<0.001
Hypertension (yes *v.s.* no)	0.00(-1.41,1.42)	0.99	--	--
Diabetes mellitus (yes *v.s.* no)	-2.19(-3.23,-1.14)	<0.001	--	--
Heart disease (yes *v.s.* no)	-0.81(-2.11,0.50)	0.22	--	--
Body mass index (per kg/m^2)	-0.11(-0.23,0.01)	0.07	--	--
Health education (per session)	0.09(0.04,0.14)	<0.001	--	--
CKD duration (per year)	0.17(0.10,0.23)	<0.001	0.16(0.10,0.22)	<0.001
iCKD usage (yes *v.s.* no)	3.05(1.93,4.16)	<0.001	1.18(0.06,2.30)	0.04
Blood urea nitrogen (per mg/dl)	0.01(-0.01,0.03)	0.29	--	--
eGFR (per $ml/min/1.73m^2$)	0.00(-0.02,0.02)	0.90	--	--
Log-formed glycated hemoglobin	-1.23(-1.75,-0.71)	<0.001	-0.87(-1.35,-0.38)	<0.001
Hemoglobin (per g/dl)	0.13(-0.13,0.39)	0.32	--	--
Albumin (per g/dl)	1.59(0.17,3.01)	0.02	--	--
Uric acid (per mg/dl)	-0.07(-0.41,0.26)	0.67	--	--
Cholesterol (per mg/dl)	0.01(-0.00,0.02)	0.10	--	--
Log-formed triglyceride	-1.13(-3.33,1.06)	0.31	--	--
Log-formed urine protein/creatinine ratio	-0.40(-1.30,0.50)	0.37	--	--

Abbreviations: CKD, chronic kidney disease; eGFR, estimated glomerular filtration rate
